# Validation of internal control for gene expression study in soybean by quantitative real-time PCR

**DOI:** 10.1186/1471-2199-9-59

**Published:** 2008-06-23

**Authors:** Bo Jian, Bin Liu, Yurong Bi, Wensheng Hou, Cunxiang Wu, Tianfu Han

**Affiliations:** 1 School of Life Sciences, Lanzhou University, Lanzhou, Gansu 730000, PR China; 2 The National Key Facility for Crop Gene Resources and Genetic Improvement (NFCRI), Institute of Crop Sciences, The Chinese Academy of Agricultural Sciences, Beijing 100081, PR China

## Abstract

**Background:**

Normalizing to housekeeping gene (HKG) can make results from quantitative real-time PCR (qRT-PCR) more reliable. Recent studies have shown that no single HKG is universal for all experiments. Thus, a suitable HKG should be selected before its use. Only a few studies on HKGs have been done in plants, and none in soybean, an economically important crop. Therefore, the present study was conducted to identify suitable HKG(s) for normalization of gene expression in soybean.

**Results:**

All ten HKGs displayed a wide range of Ct values in 21 sample pools, confirming that they were variably expressed. GeNorm was used to determine the expression stability of the HGKs in seven series sets. For all the sample pools analyzed, the stability rank was *ELF1B*, *CYP2 *> *ACT11 *> *TUA *> *ELF1A *> *UBC2 *> *ACT2/7 *> *TUB *> *G6PD *> *UBQ10*. For different tissues under the same developmental stage, the rank was *ELF1B*, *CYP2 *> *ACT2/7 *> *UBC2 *> *TUA *> *ELF1A *> *ACT11 *> *TUB *> *G6PD *> *UBQ10*. For the developmental stage series, the stability rank was *ACT2/7*, *TUA *> *ELF1A *> *UBC2 *> *ELF1B *> *TUB *> *CYP2 *> *ACT11 *> *G6PD *> *UBQ10*. For photoperiodic treatments, the rank was *ACT11*, *ELF1B *> *CYP2 *> *TUA *> *ELF1A *> *UBC2 *> *ACT2/7 *> *TUB *> *G6PD *> *UBQ10*. For different times of the day, the rank was *ELF1A*, *TUA *> *ELF1B *> *G6PD *> *CYP2 *> *ACT11 *> *ACT2/7 *> *TUB *> *UBC2 *> *UBQ10*. For different cultivars and leaves on different nodes of the main stem, the ten HKGs' stability did not differ significantly. ΔCt approach and 'Stability index' were also used to analyze the expression stability in all 21 sample pools. Results from ΔCt approach and geNorm indicated that *ELF1B *and *CYP2 *were the most stable HKGs, and *UBQ10 *and *G6PD *the most variable ones. Results from 'Stability index' analysis were different, with *ACT11 *and *CYP2 *being the most stable HKGs, and *ELF1A *and *TUA *the most variable ones.

**Conclusion:**

Our data suggests that HKGs are expressed variably in soybean. Based on the results from geNorm and ΔCt analysis, *ELF1B *and *CYP2 *could be used as internal controls to normalize gene expression in soybean, while *UBQ10 *and *G6PD *should be avoided. To achieve accurate results, some conditions may require more than one HKG to be used for normalization.

## Background

Gene expression analysis is becoming much more prevalent since it promotes our understanding of biological processes. Compared with the traditional methods for transcript analysis including Northern blot, RNase protection analysis, *in situ *hybridization and semi-RT-PCR, the fluorescence-based qRT-PCR has recently been considered as the most reliable method for the detection of mRNA [1] because of its high sensitivity, no post-PCR processing [2], and wide dynamic range [3], which allows a straightforward comparison between RNAs that differ widely in their abundance. Furthermore, it is easy to use, allows high throughput production of data and eliminates the need for radioactive isotopes [4]. Moreover, it is especially suitable when only a small number of cells are available. Although qRT-PCR is frequently used due to these advantages, some disadvantages may include variations between samples which may differ in the amount and quality of starting material, RNA preparation, cDNA synthesis, dilutions and pipetting[5]. Normalizing a target gene to the HKGs makes qRT-PCR reliable by minimizing the variations.

The HKGs, which are referred to as internal controls or reference genes, are presumed to have constant expression level among different tissues and at all developmental stages, regardless of the experimental conditions or treatments. Additionally, the HKG and target gene should have similar transcript levels to avoid analytical problems [6]. Commonly used HKGs are cellular maintenance genes, which regulate basic and ubiquitous cellular functions [7], such as components of the cytoskeleton (actins), glycolytic pathway (glyceraldehyde-3-phosphate dehydrogenase (GAPDH)), protein folding (cyclophilin), synthesis of ribosome subunits (rRNA), electron transporter (succinate dehydrogenase complex, SDH), protein degradation (ubiquitin), etc. These genes are supposed to have constant expression levels between different samples, and are frequently used as a normalizer without proper validation. However, recent studies show that the transcriptional levels of these HKGs are not always stable, and that no single HKG has a constant level under all experimental conditions [8-10]. A recent study even suggests that such a 'foolproof' gene does not exist [11]. The reason for this expression variability may be that the HKGs not only take part in the basic cell metabolism but also participate in other cellular process [12,13]. Therefore, selecting a suitable HKG(s) which has a constant expression level in certain experimental conditions for normalization is crucial for getting accurate results in gene expression studies.

Recently, many procedures have been constructed to find the best suitable HKG(s) in a set of samples, such as geNorm [11], NormFinder [14], ΔCt approach [15] and 'Stability index' [16]. For example, using geNorm, *YWHAZ*, *GAPD *and *SDHA *were found to be the most stable HKGs across the examined embryonic stages in bovine pre-implantation embryos, while the commonly used *ACTB *was variably expressed [17]. By comparing the expression results of the non-stimulated tissues and leucocytes from Atlantic salmon (Salmo salar L.) using the Normfinder program, it was shown that *EF1-alpha *was the most stably expressed gene [18]. Using the ΔCt approach, *GAPDH *was found to be the most suitable HKG for expression studies in reticulocytes while the commonly used *B2M *should be avoided [15]. *UBQ *and *TUA *were selected as reference genes to normalize gene expression in a single female poplar hybrid clone (*P. trichocarpa *× *P. deltoidies*) using the 'Stability index' [16].

Nevertheless, many studies on HKGs selection refer to human or animal tissue. As far as is known, only a few have been focused on plants such as rice [19-21], poplar [16], potato [22] and *Arabidopsis thaliana *[23]. Moreover, there is no report on soybean (*Glycine max *[L.] Merr.), a very important crop and a model plant in the early studies of photoperiodism [24,25].

Photoperiod controls several responses throughout the plant life cycle, such as germination [26], flowering induction [25], post-flowering development [27,28], maturity, dormancy [29], and yield formation [30,31]. The photoperiodic control of flowering in *Arabidopsis*, a long-day (LD) plant, is a hot topic attracting many scientists to enter this field and helping to better understand the processes involved [32-35]. However, there is little known about the mechanism in soybean, a typical short-day (SD) plant. Thus, the understanding of some key genes' expression patterns will help illuminate the mechanism involved in this process. Different cultivars of soybean may have different sensitivity to photoperiod; studies of the expression pattern of key genes in the photoperiodic pathway may also help elucidate what leads to the varietal difference.

In the present study, the expression profiles of ten HKGs, including *ACT11*, *ACT2/7*, *TUA*, *TUB*, *UBC2*, *CYP2*, *G6PD*, *ELF1A*, *ELF1B *and *UBQ10*, were studied during the development of *Zigongdongdou *(ZGGG), a late-maturing soybean (*Glycine max *[L.] Merr) cultivar, under LD and SD conditions. The expression patterns of the ten HKGs were also detected in soybean cultivar Heihe No. 27 (HH27), Zhonghuang No. 24 (ZH24) and Suinong No. 14 (SN14). GeNorm, ΔCt approach and 'Stability index' were used to assess the value of ten HKGs as suitable internal control(s) for soybean gene expression studies.

## Results

### Expression profiling of HKGs

**Figure 1:**
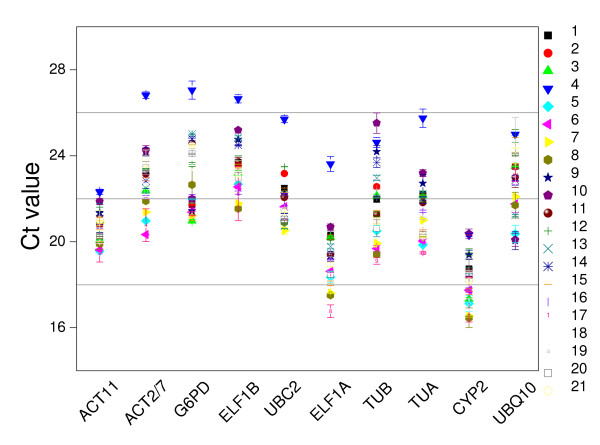
**The transcriptional profiles of individual HKGs in absolute Ct values over all RNA samples**. The tissues used for this analysis were listed in Table 4. The number indicated the corresponding sample.

A qRT-PCR assay, based on SYBR Green detection, was designed for the transcriptional profiling of ten commonly used HKGs (*ACT11*, *ACT2*, *G6PD*, *ELF1B*, *UBC2*, *ELF1A*, *TUB*, *TUA*, *CYP2 *and *UBQ10*) in soybean. In order to select a reliable set of HKGs, all PCR assays were done in triplicate. To make the comparison among each PCR run reliable, all the Ct values were determined at the threshold fluorescence value of 0.2, and three fixed PCR reactions were performed in every PCR run to make the data from each PCR run comparable. The Ct value was used to analyze the transcriptional levels of HKGs. This approach was a simplified way to give an overview of the abundance of the genes in the samples [36]. The ten HKGs showed a relatively wide range of Ct values from the lowest mean Ct value (18.22) in *CYP2*, to the highest (23.50) in *ELF1A *in all tested sample pools in soybean. Individual HKGs had different expression levels across all the sample pools tested. *ACT11 *and *CYP2 *showed the smallest gene expression variation (below 4 cycles), while *ACT2/7*, *ELF1A*, *TUB *and *TUA *had the highest expression variation (above 6 cycles) as shown in Figure 1. The wide expression range of the ten tested HKGs confirmed that no single HKG had a constant expression under these conditions in soybean. Obviously, it is necessary to select a suitable HKG to normalize gene expression under a certain condition.

### GeNorm analysis

**Figure 2:**
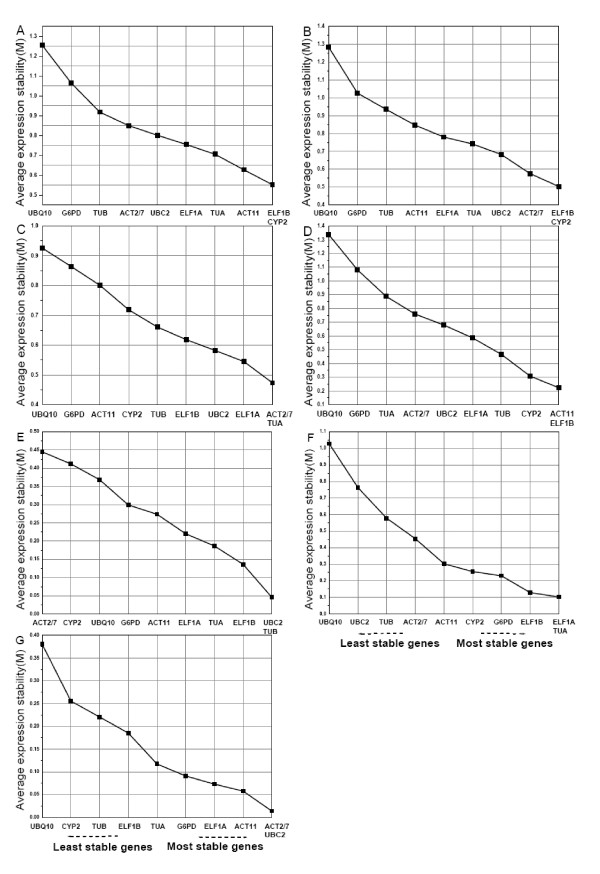
**Average expression stability and ranking of ten HKGs as calculated by geNorm**. Expression stability and ranking of ten HKGs calculated with geNorm in all 21 sample pools (A), different tissues at the same developmental stage (B), developmental series (C), photoperiod treatments (D), different cultivar (E), different time of the day series (F), leaves located on different nodes on the main stem (G). A lower average expression stability M indicates more stable expression.

Gene expression stability (M) of these ten HKGs in various tissue samples under different conditions was measured by geNorm as described by Vandesompele *et al*. [11]. This approach relies on the principle that the expression ratio of two ideal HKGs is constant in all the samples, independent of the experimental conditions and cell-types. Genes with the lowest M have the most stable expression, while the highest M value indicates the least stable expression [11]. We analyzed data under seven sets. As shown in Figure 2A, when all the 21 samples were taken together, the average expression stability value (M) of *ELF1B *and *CYP2 *was lowest, and that of *UBQ10 *was highest, suggesting that *ELF1B *and *CYP2 *had the most stable expression and that *UBQ10 *was expressed most variably. The results remained very similar in the different tissues under the same developmental stage series, with the lowest M value for *ELF1B *and *CYP2 *(Figure 2B). *UBQ10 *remained the least stable gene, while *ACT2/7 *and *TUA *were the ones with the lowest M, indicating that they were stably expressed in the developmental series of soybean (Figure 2C). In the photoperiodic treatment, *ACT11 *and *ELF1B *were the most stable genes, while *UBQ10 *still was the most variable one (Figure 2D). In the different cultivar series, the M value was least for *UBC2 *and *TUB *followed by *ELF1B*, *TUA*, *ELF1A*, *ACT11*, while *ACT2/7 *was the least stable HKG (Figure 2E). In the different time of the day series, *ELF1A *and *TUA *were expressed much more stably than the other eight HKGs, while *UBQ10 *continued to be the most variable one (Figure 2F). Since the HKGs *UBQ10 *and *ACT11 *showed variable expression profiles in a semi-RT-PCR comparison of the unifoliate leaves and a leaf mixture containing all the leaves (data not shown), the transcriptional expression of the ten HKGs was studied in this series. Results from geNorm analysis showed *UBC2 *and *ACT2/7 *were the least variable ones among the ten tested HKGs, while *UBQ10 *was still the most variable one. However, the difference of M value between the less stable HKGs (*ACT11*, *ELF1A*, *G6PD *and *TUA*) was minimal (Figure 2G).

**Figure 3:**
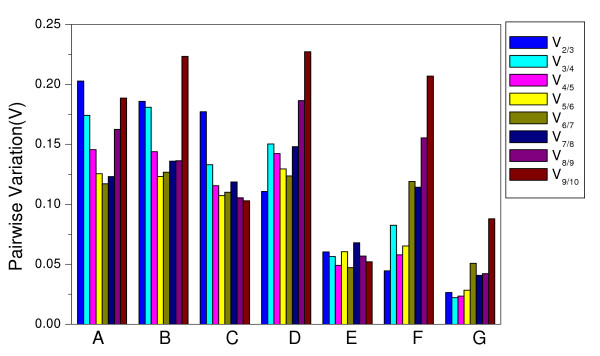
**Determination of the optimal number of HKG for normalization by pairwise variation using geNorm**. Pairwise variation (V) to determine the optimal number of HKG(s) for accurate normalization in all 21 sample pools (A), different tissues at the same developmental stage (B), developmental series (C), photoperiod treatment (D), different cultivars (E), different time of the day series (F), leaves on different nodes on the main stem (G).

For some experimental setups, using a single HKG for normalization is appropriate [20], while, for other ones, there may be no single HKG suitable as a reliable internal control [11]. Therefore, the requirement of two or more HKGs for accurate normalization is necessary. The optimal number of HKGs necessary for reliable normalization is defined by a normalization factor (NF) which is determined by the geNorm software. The pairwise variations V_n_/V_n+1 _between two sequential normalization factors (NF_n _and NF_n+1_) are used to determine the necessity of adding the next HKG for reliable normalization [11]. As shown in Figure 3(D,E,F,G), the two most stable HKGs were found to be optimal for the accurate normalization with a pairwise variation value much lower than the cut-off value of 0.15 suggested in [11]. It was apparent that the addition of the third HKG for normalization would have no significant effect on pairwise variation in the four series as shown in Figure 3A. When all 21 samples were taken together, the pairwise variation V_2/3 _was higher than 0.15 (0.203), as was V_3/4 _(0.174). This indicated the addition of the fourth HKG was necessary to normalize gene expression. This situation was similar for the series with different tissues under the same developmental stage. The pairwise variation V_2/3 _and V_3/4 _were 0.185 and 0.181, respectively, both higher than 0.15 (Figure 3B). When looking at the developmental stages, the pairwise variation V_2/3 _was 0.177, while V_3/4 _was 0.133, so the three HKGs (*ELF1A*, *ACT2/7 *and *TUA*) were sufficient for accurate normalization. When evaluating all the pairwise variation, the least stable HKG was *UBQ10 *followed by *G6PD *as they significantly increased the pairwise variation during the whole assay by increasing the V value as shown in Figure 3.

### 'Stability index' assay

A 'Stability index' assay was first used to select suitable internal controls during the development of poplar [16]. In the current study, the expression stability rank of the ten HKGs in soybean was detected according to the 'Stability index'. As shown in Table 1, when all 21 sample pools were taken together, *ACT11 *had the lowest stability index and was the most stable HKG. The expression stability rank was as follows: *ACT11 *> *CYP2 *> *UBC2 *> *ELF1B *> *ACT2/7 *> *G6PD *> *UBQ10 *> *TUB *> *TUA *> *ELF1A*.

**Table 1:** Summary of statistics measuring stability of HKG expression

Gene	Mean Ct^a^	StdDev^b^	Slope^c^	Intercept^d^	CV%^e^	Stability index^f^
*ACT11*	18.63	0.74	0.03	20.97	3.57	0.11
*CYP2*	18.22	1.09	0.02	18.46	5.99	0.13
*UBC2*	21.91	1.17	0.03	21.91	5.33	0.16
*ELF1B*	23.50	1.21	0.05	24.01	5.16	0.24
*ACT2/7*	22.82	1.42	0.05	23.33	6.21	0.28
*G6PD*	22.91	1.68	0.04	22.45	7.34	0.31
*UBQ10*	22.85	1.53	0.06	22.14	6.68	0.43
*TUB*	21.77	1.74	0.09	22.80	7.99	0.72
*TUA*	21.47	1.42	0.12	22.86	6.63	0.80
*ELF1A*	19.25	1.43	0.12	20.58	7.45	0.90

^a^Data based on analysis of raw Ct value. Genes were ordered, top to bottom, from those tending to show the highest stability to those showing the lowest, based on the stability index. ^b^StdDev among all the tissue samples tested. ^c^Slope of regression of gene means. ^d^Intercepts were also given for the estimated regression lines. ^e^Coefficient of variation (CV) (StdDev divided by mean multiplied by 100). ^f^Stability index was the product of CV and slope (multiplication of columns 4 and 6). Genes with the lowest stability index usually provide the best control.

### ΔCt analysis

The ΔCt approach was employed by Silver et al.[15] to select the most suitable HKG in reticulocytes. If the ΔCt value between two HKGs does not change when analyzed in all the samples, it means either both genes have stable expression patterns or they are co-regulated among those samples. However, the fluctuation in ΔCt means that at least one of them was variably expressed. Introduction of a third, fourth, or fifth gene into the comparisons will provide more information on which pairs show less variability and hence which gene(s) has stable expression among the samples tested. It is easy for large panels of genes to be compared with one another and then selected or eliminated on the basis of ΔCt. Ultimately, an appropriate HKG can be selected for a particular experimental system [15].

The expression stability of HKGs was measured by the ΔCt value and standard deviation (StdDev) in the present study as described in [15]. Taking all ten HKGs into account and comparing all the possible combinations, their expression stability was determined. As shown in Additional File 1, Figure 4A and 4B, when *ELF1B *and *CYP2 *were compared with the other nine HKGs in their respective gene panels, the mean StdDev was 1.00 and 1.04, respectively, indicating that *ELF1B *was the most stable one among the ten HKGs analyzed in all the 21 sample pools, followed by *CYP2*. In contrast, when *UBQ10 *and *G6PD *were compared to the other nine HKGs in their respective gene panels, they tended to be associated with the greatest amount of deviation in ΔCt value (the mean StdDev was 1.84 and 1.50, respectively), which meant that *UBQ10*, followed by *G6PD*, were the least stable HKGs in all the 21 samples tested. *UBQ10 *should be avoided when doing gene expression analysis because of its high deviation in ΔCt value when compared to the other nine HKGs (the greatest amount was 2.44 to *TUB*, and the least was 1.47 to *UBC2*). *ELF1A*, *ACT11*/*TUA*, *TUB*/*ACT2/7 *and *UBC2 *all showed intermediate levels of ΔCt deviation (the mean StdDev was 1.06, 1.07, 1.07, 1.08, 1.08, 1.15, 1.50 and 1.84, respectively), indicating intermediate stability. Overall rankings were as follows: *ELF1B*, *CYP2*, *ELF1A*, *ACT11*/*TUA*, *TUB*/*ACT2/7*, *UBC2*, *G6PD*, and finally *UBQ10*.

**Figure 4:**
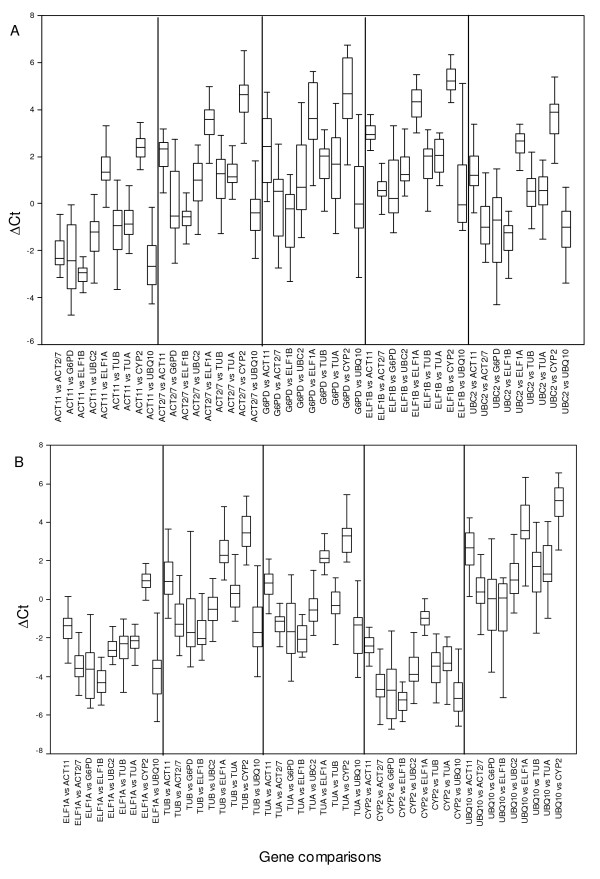
**ΔCt method for HKG selection**. ΔCt variability in HKG comparisons were shown as medians (lines), 25th percentile to the 75th percentile (boxes) and ranges (whiskers) for all the 21 sample pools. A. Comparisons of the completely possible sets of HKGs which included *ACT11*, *ACT2/7*, *G6PD*, *ELF1B*, *UBC2*; B. Comparisons of the completely possible sets of HKGs, which included *ELF1A*, *TUB*, *TUA*, *CYP2 *and *UBQ10*.

### *GmBFT *expression

**Figure 5:**
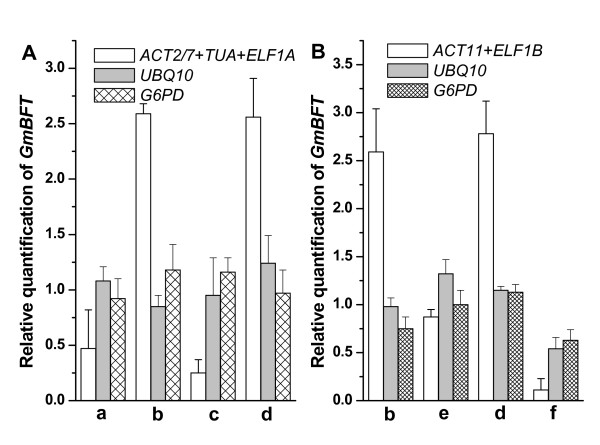
**Relative quantification of *GmBFT *expression using different HKGs as internal controls under different developmental stages and photoperiod conditions**. Relative quantification of *GmBFT *expression was detected using two or three of the most stable HKGs or the most variable HKGs selected by geNorm as internal controls. The geometric average of *ACT2/7*, *TUA *and *ELF1A*, *UBQ10 *and *G6PD *were used as internal controls for developmental stage (A), the geometric average of *ACT11 *and *ELF1B*, *UBQ10 *and *G6PD *were used as internal controls for photoperiod treatment (B). a, SD 1-day leaves; b, SD 25-day leaves; c, SD 1-day shoot tips; d, SD 25- day shoot tips; e, LD 25-day leaves; f, LD 25-day shoot tips.

The relative expression level of *GmBFT *(*Glycine max *brother of FT and TFL1) [37], an ortholog of *Arabidopsis BFT *[38], was detected to validate the HKGs selected in the present study under certain conditions according to the geNorm manual. The geometric average of the most stable HKGs (*ACT2/7*, *TUA *and *ELF1A*) in the developmental stage series selected by geNorm was used as an internal control. The relative expression of *GmBFT *increased after a 25-day SD treatment in unifoliate leaves, being 5.5-fold higher than that at 1-day SD treatment (Figure 5A b and a, Table 2 b and a). Similarly, *GmBFT *expression level was also higher in shoot tips after a 25-day SD treatment compared to the 1-day SD treatment (Figure 5A d and c, Table 2 d and c). In the same way, using the geometric average of the most stable HKGs (*ACT11 *and *ELFIB*) in the photoperiodic treatment selected by geNorm as an internal control, it was found that the relative expression of *GmBFT *expression in both unifoliate leaves and shoot tips at 25-day under SD treatment was higher than that under LD treatment. The expression level of *GmBFT *in unifoliate leaves under 25-day SD treatment was 2.98 fold higher than that under 25-day LD treatment (Figure 5B b and e, Table 3 a and b). Likewise, *GmBFT *expression level in shoot tips under 25-day SD treatment was 25.3 fold higher than that under 25-day LD treatment (Figure 5B d and f, Table 3 c and d). As shown previously by geNorm in the present study, *GAPD *and *UBQ10 *were the most variable HKGs for the developmental stage and photoperiodic treatment series. The relative expression of *GmBFT *in unifoliate leaves and shoot tips was also detected using the two HKGs as internal controls and no significant difference was found.

**Table 2:** Relative expression of *GmBFT *at different developmental stages

Tissue sample	Relative expression of *GmBFT *using different HKG(s) as internal controls (mean ± StdDev)		
	
	*ACT2/7+TUA+ELF1A*	*UBQ10*	*G6PD*
a	0.47 ± 0.35	1.08 ± 0.13	0.92 ± 0.18
b	2.59 ± 0.09	0.85 ± 0.10	1.18 ± 0.23
c	0.25 ± 0.12	0.95 ± 0.34	1.16 ± 0.13
d	2.56 ± 0.35	1.24 ± 0.25	0.97 ± 0.21

a, SD 1-day leaves; b, SD 25-day leaves; c, SD 1-day shoot tips; d, SD 25- day shoot.

**Table 3:** Relative expression of *GmBFT *under different photoperiodic treatments

Tissue sample	Relative expression of *GmBFT *using different HKG(s) as internal controls (mean ± StdDev)		
	
	*ACT11+ELF1B*	*UBQ10*	*G6PD*
a	2.59 ± 0.45	0.98 ± 0.09	0.75 ± 0.12
b	0.87 ± 0.08	1.32 ± 0.15	1.00 ± 0.15
c	2.78 ± 0.34	1.15 ± 0.04	1.13 ± 0.08
d	0.11 ± 0.12	0.54 ± 0.12	0.63 ± 0.11

a, SD 25-day leaves; b, LD 25-day leaves; c, SD 25- day shoot tips; d, LD 25-day shoot tips.

## Discussion

qRT-PCR has become a powerful tool for gene expression analysis because of its high throughput, sensitivity and accuracy [1,4]. The use of suitable HKGs to normalize the variation made by RNA preparation, cDNA synthesis or PCR processing would make the results more reliable. In order to select suitable HKG(s) for normalization, many procedures such as geNorm [11], Normfinder [14], ΔCt approach [15] and 'Stability index' [16] have been used. Since all methods mentioned above are based on the Ct value, which is determined mostly by the quantity of cDNA [39], the prerequisite for selecting a set of reliable HKG(s) is based on the equal input cDNA when doing qRT-PCR. In early studies, the most commonly used method to measure the input cDNA was by a spectrophotometer such as NanoDrop ND-1000 [20,40,41]. Considering the importance of input cDNA, more than one method should be used to detect its quantity and quality to ensure the cDNA equality for each PCR run in order to get reliable results for HKG(s) expression stability analysis. In the present study, cDNA was verified by measurements on both the ND-1000 and SMA3000 spectrophotometers to ensure the equality of cDNA in the PCR reactions and reliability of the results when using Ct values for analysis.

When gene expression stability in soybean was analyzed by geNorm, the most stable genes in the seven series were different as shown in Figure 3. In all seven series analyzed, *ELF1B *was the most stable HKG. *UBQ10 *and *G6PD *were the most variable ones, so these genes should be avoided as internal controls when doing gene expression studies in soybean. Our findings were in accordance with the result that *UBQ10 *exhibited the least stable expression in different tissues or cell types at different developmental stages in rice [20]. Similarly, in the development of grape berry, *UBQ10 *was not the recommended HKG for normalization [42]. However, in an earlier study in *Arabidopsis*, *UBQ10 *showed highly stable expression [23]. An ubiquitin tag is not only used to mark particular proteins for proteolytic elimination, but can also have non-proteolytic functions [43] which may lead to the variable expression of ubiquitin in different plants. *G6PD *was suggested to be an inappropriate internal control in qRT-PCR studies of estrogens effects in fish [44] and it also showed significant differences in expression between malignant and nonmalignant pairs (at least p < 0.04) of human bladder cancer [45]. There are few published works using *G6PD *as an internal control. This may be because *G6PD *not only acts as a component of the glycolytic pathway but also participates in other processes as well. Thus, the expression profile of *G6PD *might change according to the corresponding experimental conditions. In the present study, *TUA *was found to be one of the most stable HKGs with the lowest M value in the developmental series in soybean. This result was consistent with an earlier study in poplar with *TUA *as one of the most stable HKGs [16]. Other most commonly used HKGs, like *TUB*, displayed an unacceptably high variable expression pattern limiting its use as an internal control except in the different cultivar series where all the other HKGs showed relatively stable expression. Taken together, these results suggested that these HKGs were regulated differently in each plant species and may exhibit differential expression patterns. Therefore, a HKG with stable expression in one organism may be not suitable to normalize gene expression in another organism under a given set of conditions and thus needs to be validated before its use.

To further verify the suitability of HKGs selected in the present study, *GmBFT *expression levels were detected at different developmental stages and under different photoperiodic conditions in soybean (Figure 5). In unifoliate leaves, *GmBFT *expression after a 25-day SD treatment was significantly higher than after a 1-day SD treatment, and was also higher than after a 25-day LD treatment. The case was similar in the shoot tips of soybean using HKGs selected in corresponding conditions as internal controls. The fact that this result is in accordance with earlier work by Sun et al. [37] means that the HKGs identified in this study are suitable at various development stages and photoperiodic conditions. The relative expression of *GmBFT *was also analyzed using *UBQ10 *and *G6PD *as internal controls (Figure 5). No significant expression difference of *GmBFT *was observed in unifoliate leaves at different developmental stages or under different photoperiodic conditions. The result was similar in shoot tips. Obviously, *UBQ10 *and *G6PD *are not suitable HKGs to normalize gene expression in soybean under such conditions.

The ΔCt approach [15] and 'Stability index' [16] were also used to analyze gene expression stability in all 21 sample pools in soybean to compare the accuracy of these three methods. Results obtained from the ΔCt method were very similar to that from the geNorm analysis. *ELF1B *was the most stable HKG followed by *CYP2*, while *UBQ10 *was the most variable HKG followed by *G6PD*. When 'Stability index' was used to measure the stability of HKG(s), results changed, with *ACT11 *being the most stable HKG followed by *CYP2*, while *TUA *and *ELF1A *became the least stable ones in all the 21 sample pools in soybean. An explanation might be that the 'Stability index'did not take the PCR efficiency into account, which played an important role in the data analysis. Thus, in order to get a reliable result by only comparing Ct deviation for the individual genes in the tested tissue samples, the HKGs analyzed should have similar PCR efficiency.

Although gene expression stability analyzed by the ΔCt approach was similar to that by geNorm, it was still not the first choice to get accurate normalization especially for the research which can get enough samples. The ΔCt method can be used to detect the most stable HKG as geNorm provides; however, it could not provide the number of HKGs necessary for accurate normalization. Some studies may require more than one HKG to be included, and using the single most stable HKG for normalization might not get the most accurate result. Indeed, as shown in Figure 3A, when all 21 sample pools were analyzed by geNorm, the geometric average of four HKGs (*ELF1B*, *CYP2*, *ACT11 *and *TUA*) was recommended as the normalization factor to get accurate results in soybean. However, the analysis by ΔCt method only indicated that the most stable HKG was *ELF1B*, and it could not be used to find out the optimal number of HKGs. Thus, gene expression accuracy might be undermined if the result is only based on *ELF1B*. It is true that the ΔCt method is useful for validating the most stable HKG in some specific tissue samples or cell types for which it is difficult to obtain enough material, such as reticulocytes [15]. However, for material where RNA samples are easy to obtain, such as plants, geNorm is recommended because it is easy to determine the optimal number of stable HKGs for accurate normalization.

Selection of suitable HKG(s) is necessary for accurate gene expression, but it is quite expensive and time-consuming. To avoid the additional expense and labor of using multiple internal control genes, a potential strategy suggested by Brunner *et al*. [16] was to design a PCR primer pair which could amplify two or more members of a control gene family. However, for two members of the same HKG family, their expression pattern and stability might vary. Therefore, results may be questionable for gene expression based on these two HKGs. For example, *UBQ5 *was one of the most suitable HKGs in a given set of tissue samples in rice, while *UBQ10 *expressed variably [20]. A similar situation was observed for the actin gene family in the developmental stage series in the present study. *ACT2/7 *was stably expressed, while *ACT11 *showed variable profiling. Thus, using primer pair sets to amplify two or more members of a HKG family for qRT-PCR may not be recommendable. After all, the accuracy of the results should always be given first priority.

The photoperiod plays an important role throughout the life cycle of soybean [27,28,46]. The sensitivity of different cultivars of soybean to photoperiod is quite variable. Understanding the mechanisms involved in this process will be beneficial in the molecular breeding of soybean. In the present study, the most stable HKG in the different cultivar series under the same photoperiodic conditions and developmental stage was *UBC2*, whereas *ACT2/7 *was the most variable one. The difference of average expression stability among the ten HKGs in this series was smaller than that in the other series. Thus, the gene expression would be similar using any of the ten HKGs as internal controls when studying different cultivar. It would be helpful to understand the mechanisms involved in differential sensitivities to photoperiod for different cultivars.

## Conclusion

A large number of studies have been carried out concerning the validation of HKG(s) in many different tissue samples and cell types. However, there is no correlative report in soybean, a SD dicot. Our data showed the variable expression profiles of ten commonly used HKGs in different tissue samples and under different photoperiodic conditions in soybean. Based on geNorm and ΔCt methods, *ELF1B *and *CYP2 *appears to be the most suitable HKGs to normalize gene expression during the development of soybean, while *UBQ10 *and *G6PD *seems to be unsuitable as reference genes. Under some conditions, more than one HKG should be used as internal controls to normalize gene expression in soybean in order to get the most reliable results.

## Methods

### Sample collection and RNA extraction

ZGGG, a soybean (*Glycine max *[L.] Merr.) cultivar from Sichuan Province, South China, was used as the main material. This cultivar is late maturing and sensitive to photoperiod [27,28]. HH27, a soybean cultivar from Heilongjiang Province, which is not sensitive to photoperiod; ZH24, a soybean cultivar from Beijing and SN14, another soybean cultivar from Heilongjiang, were also used as the materials. After the unifoliate leaves expanded, the seedlings were transferred to LD (16 h light/8 h dark) and SD (12 h light/12 h dark) conditions, respectively [28]. The samples were collected and then frozen in liquid N_2 _and stored at -80°C until RNA extraction. All the sample pools used for this research were provided in Table 4 and each pool contained at least 30 seedlings.

**Table 4:** Soybean tissues used for gene expression analysis

No.	Cultivar	Organ	Photoperiod	Time after photoperiodic treatment for seeding
1	ZGDD	Root	LD	1d
2	ZGDD	Root	LD	25d
3	ZGDD	Root	SD	25d
4	ZGDD	Stem	SD	1d
5	ZGDD	Stem	SD	25d
6	ZGDD	Stem	LD	25d
7	ZGDD	Shoot tip	SD	25d
8	ZGDD	Shoot tip	SD	1d
9	ZGDD	Root nodule	SD	25d
10	ZGDD	Root nodule	LD	25d
11	ZGDD	Leaf	SD	25d
12	ZGDD	Leaf	LD	25d
13	ZGDD	Leaf	LD	2 h
14	ZGDD	Leaf	LD	6 h
15	ZGDD	Leaf	LD	10 h
16	ZGDD	Flower	SD	25d
17	ZGDD	Pod	SD	32d
18	ZGDD	Leaf	LD	1d
19	HH27	Leaf	LD	1d
20	ZH24	Leaf	LD	1d
21	SN14	Leaf	LD	1d

Total RNA was extracted using Trizol (Invitrogen) according to the Manufacture's Instruction with little modification. One more chloroform extraction step was added to the RNA extraction process. RNA was quantified by the absorbance at OD_260 _using NanDrop ND-1000 spectrophotometer. The absorbance ratio at OD_260/280 _and OD_260/230 _were used to assess the purity of all the RNA samples. Only RNA samples with OD_260/280 _ratio (protein contamination) between 1.8–2.0 and OD_260/230 _(organic pollutant) higher than 2.0 was used for the further analysis. RNA integrity was verified by 2% agar gel electrophoresis and ethidium bromide staining. The samples with 25S/18S ribosomal RNA between 1.5–2.0 and absence of smears were used for the following experiment.

### cDNA synthesis and quantification

Before cDNA synthesis, 5 μg total RNA was treated with RQ1 RNase-free DNase (Promega) according to the Manufacture's Instruction to ensure no DNA contamination, and then cDNA synthesis was carried out with the purified RNA using the SuperScript III First-Strand Synthesis System (Invitrogen) following the instruction. The RT reaction was performed using Mastercycler Gradient (Eppendorf). Briefly, 1 μg RNA, 50 μM oligo_dT_(20) and 10 mM dNTP mix were added together to incubate at 65°C for 5 min, then placed on ice for at least 1 min. After that, 2 μl 10 × RT buffer, 1 μl 25 mM MgCl_2_, 2 μl 0.1 M DTT, 40 U RNaseOUT and 200 U SuperScript III were added and then incubated at 50°C for 50 min. The RT reaction was terminated by incubating at 85°C for 5 min and the residual RNA was removed by incubated at 37°C for 20 min with the addition of 1 μl RNaseH. After cDNA was synthesized, it was used as the template for PCR amplification. This amplification was made using primer pair sets which span an intron to detect DNA contamination.

cDNA was 2× diluted before quantification and the quantity and quality of input cDNA were determined by the SMA3000 and NanDrop ND-1000 spectrophotometer to make sure the cDNA amount for each PCR run is equal. For each method, the measurement was done in duplicate. The slope of the regression line of the concentrations measured with both methods did not differ indicating the equality of cDNA measurement with both methods for the qRT-PCR reaction (Yconc.ND-1000 = 1.0444 × Xconc.SMA3000+0.5443; n = 21; r = 0.9763). To reduce the system error, all the cDNA was diluted to about 2.3 ng/μl, so there were 20 ng/8.8 μl cDNA for the real-time RT-PCR reaction. All the cDNA were stored at -20°C until PCR.

### Selection of soybean sequences, primer design and PCR optimization

Sequences for the primer design were selected according to Brunner *et al*. [16] to identify soybean homologs for genes which are commonly used as internal controls. The soybean EST database [47] was queried with the relevant *Arabidopsis *protein using TBLASTN. Selected soybean ESTs were then used to query the *Arabidopsis *protein database using BLASTX. Primers were designed with Primer Premier 5 [48] with melting temperature between 60–62°C, 18–20 bp and about 50% GC content. The primers were used to query soybean EST database with BLASTN to ensure the specificity for the selected gene family member (Table 5). Since there is little known about the genomic DNA sequence of soybean, alignments were made with DNA sequence of relevant orthologs in *Arabidopsis *before primer design to ensure the primer pairs span at least one intron. The primer sequence, primer positions (indicating that the primers span an intron) and amplicon length were provided in Table 6. Before qRT-PCR, the primer pairs were tested by standard PCR reaction with Mastercycler Gradient (Eppendorf) to find out the best suitable conditions. Amplicons of expected size were verified by 2% agarose gel electrophoresis and ethidium bromide staining.

**Table 5:** Description of soybean genes for qRT-PCR

Name^a^	Soybase Accession Number	*Arabidopsis *homolog locus^b^	*Arabidopsis *locus description	Function	BLASTX score/E Value
*ACT11*	TC204137	AT3G12110	Actin11	Cytoskeletal structural protein	740/0
*ACT2/7*	TC204150	AT5G09810	Actin2/7	Cytoskeletal structural protein	739/0
*G6PD*	TC224599	AT5G40760	Glucose-6-phosphate dehydrogenase	Glucose metabolic process	903/0
*ELF1B*	TC203623	AT5G19510	Eukaryotic elongation factor 1-beta	Translational elongation	245/2e-65
*UBC2*	TC214734	AT2G02760	Ubiquitin-conjugating enzyme E2	Ubiquitin-dependent protein catabolic process	314/5e-86
*ELF1A*	TC203954	AT5G60390	Eukaryotic elongation factor 1-alpha	Translational elongation	469/e-132
*TUB*	TC203804	AT1G50010	Beta-tubulin	Structural constituent of cytoskeleton	847/0
*TUA*	AY907702	AT5G19780	Tubulin alpha-5	Structural constituent of cytoskeleton	803/0
*CYP2*	TC224926	AT2G21130	Cyclophilin	Protein folding	283/9e-77
*UBQ10*	TC203625	AT4G05320	Ubiquitin 10	Protein binding, protein modification process	734/0
*GmBFT*	EF532597	AT5G62040	Brother of FT and TFL1 protein	Phosphatidylethanola -mine binding	231/4e-61

^a^All soybean sequences except *TUA *were ESTs based on the *Arabidopsis *proteins determined via BLASTX. ^b^Closest *Arabidopsis *homolog identified using Tair BLAST [50]. AGI protein database was queried with soybean nucleotide sequences using BLASTX.

**Table 6:** Primers and amplicons for each of the 10 HKGs and *GmBFT*

Name	Forward Primer Sequence [5'-3']	Reverse Primer Sequence [5'-3']	Amplicon Length (bp)	Primer location^a^	E(%)	R^2^
*ACT11*	CGGTGGTTCTAT CTTGGCATC	GTCTTTCGCTTCAA TAACCCTA	142	D	98.04	0.9979
*ACT2/7*	CTTCCCTCAGCA CCTTCCAA	GGTCCAGCTTTCA CACTCCAT	119	D	109.56	0.9979
*G6PD*	ACTCCTTGATAC CGTTGTCCAT	GTTTGTTATCCGCC TACAGCCT	126	D	98.59	0.9990
*ELF1B*	GTTGAAAAGCCA GGGGACA	TCTTACCCCTTGA GCGTGG	118	D	93.15	0.9883
*UBC2*	TCCCCTCACACC CTTCCTC	CCATCCCAAGGGG TGTCAT	155	D	100.60	0.9988
*CYP2*	CGGGACCAGTGTGCTTCTTCA	CCCCTCCACTACAAAGGCTCG	154	S	82.31	0.9848
*ELF1A*	GACCTTCTTCGT TTCTCGCA	CGAACCTCTCAAT CACACGC	195	D	98.21	0.9981
*TUB*	CCTCGTTCGAAT TCGCTTTTTG	CAACTGTCTTGTC GCTTGGCAT	161	S	100.84	0.9956
*TUA*	AGGTCGGAAACT CCTGCTGG	AAGGTGTTGAAGG CGTCGTG	159	S	85.75	0.9829
*UBQ10*	CGCCTCTAATCT CGCAGTTCC	GTTGTCAATGGTG TCGGAGGA	114	S	120.28	0.9663
*GmBFT*	CCAAGGGAAATT GTGAGGTA	CTACTAAAAAGCC CCACAGC	191	S	101.65	0.9985

^a^D, the two primers were located on different exons; S, the two primers were located on the same exon.

### qRT-PCR

qRT-PCR was conducted on ABI PRISM 7000 Sequence Detection System using power SYBR Green Mix (Applied Biosystems, USA). Each reaction was run in a 20 μl volume which contained 8.8 μl cDNA equal to 20 ng, 10 μl 2 × power SYBR mix, 0.6 μl each primer to a final concentration of 300 nM. All the reactions were performed as the following conditions: 2 min at 50°C, 10 min at 95°C, and 40 cycles of 10 s at 95°C, and 1 min at 60°C in 96-well optical reaction plates (Applied Biosystems, USA). To verify the specificity of the amplicon for each primer pair, a melting curve was made from 60°C to 95°C at the end of each PCR run and all the ten primer pairs amplified a single product. The PCR efficiencies showed in Table 6 for each gene was determined with the slope of a liner regression model. Each cDNA sample pool was bulked and then used as the PCR template in a range of 50, 25, 10, 5, and 2 ng [22]. The corresponding real-time PCR efficiencies were calculated according to the equation: E = 10^-1/slope ^[4].

### Data processing

Expression levels of the ten HKGs in all the sample pools were determined by the number of cycles (Ct) needed for the amplification related fluorescence to reach a specific threshold level of detection [39]. The raw Ct value obtained from ABI 7000 after each PCR run was converted into relative quantities using the PCR efficiencies for each gene according to the requirement of geNorm software [11,49] to calculate gene expression stability (M). The expression stability of the ten HKGs was also determined by the 'Stability index' [16] and ΔCt approach [15] to compare the three methods in all the 21 sample pools.

## Abbreviations

HKG, housekeeping gene; qRT-PCR, quantitative real-time PCR; *ACT11*, *actin11*; *ACT2/7*, *actin2/7*; *G6PD*, *glucose-6-phosphate dehydrogenase*; *ELF1B*, *eukaryotic elongation factor 1-beta*; *UBC2*, *ubiquitin-conjugating enzyme E2*; *ELF1A*, *eukaryotic elongation factor 1-alpha*; *TUB*, *beta-tubulin*; *TUA*, *alpha-tubulin *; *CYP2*, *cyclophilin*; *UBQ10*, *ubiquitin 10*; ZGGG, Zigongdongdou; HH27, Heihe No. 27; ZH24, Zhonghuang No. 24; SN14, Suinong No. 14; SD, short day; LD, long day; StdDev, standard deviation; CV, coefficient variation; *GmBFT*, *Glycine max brother of FT and TFL1*.

## Authors' contributions

BJ performed all the experimental procedures, data analysis, draft the manuscript and was the primary author of the manuscript. BL participated in data analysis, tables and figures drawing and manuscript revising. YB designed the study. WH participated in the experimental process and provided technical support throughout the experimental process. CW performed the sample preparation. TH supervised the study, revised the manuscript critically and gave financial support to the study.

## Supplementary Material

Additional File 1 **HKG comparisons**. Mean ΔCt values were given for the mean difference between the genes over the 21 sample pools. SteDev was given for the variation in Ct values over the 21 sample pools. Click here for file
